# Predictive Accuracy of COVID-19 World Health Organization (WHO) Severity Classification and Comparison with a Bayesian-Method-Based Severity Score (EPI-SCORE)

**DOI:** 10.3390/pathogens9110880

**Published:** 2020-10-24

**Authors:** Christophe de Terwangne, Jabber Laouni, Lionel Jouffe, Jerome R. Lechien, Vincent Bouillon, Sammy Place, Lucio Capulzini, Shahram Machayekhi, Antonia Ceccarelli, Sven Saussez, Antonio Sorgente, on behalf of EPIBASE TEAM

**Affiliations:** 1 Department of Internal Medicine, Epicura Centre Hospitalier, 7301 Hornu, Belgium; Jabber.Laouni@ulb.ac.be (J.L.); Vincent.Bouillon@ulb.ac.be (V.B.); sammy.place@epicura.be (S.P.); 2 Bayesia, 53810 Changé, France; jouffe@bayesia.com; 3 Department of Human Anatomy and Experimental Oncology, Faculty of Medicine, UMONS Research Institute for Health Sciences and Technology, University of Mons (UMons), 7000 Mons, Belgium; jerome.lechien@umons.ac.be; 4 Department of Otolaryngology-Head & Neck Surgery, CHU de Bruxelles, CHU Saint-Pierre, School of Medicine, Université Libre de Bruxelles, 1000 Brussels, Belgium; 5 Department of Otolaryngology-Head & Neck Surgery, Foch Hospital, School of Medicine, UFR Simone Veil, Université Versailles Saint-Quentin-en-Yvelines (Paris Saclay University), 92150 Paris, France; 6 Department of Cardiology, Epicura Centre Hospitalier, 7301 Hornu, Belgium; lucio.capulzinicremonini@epicura.be; 7 Department of Intensive Care, Epicura Centre Hospitalier, 7301 Hornu, Belgium; shahram.machayekhi@epicura.be; 8 Department of Neurology, Epicura Centre Hospitalier, 7800 Ath, Belgium; ceccarelli.antonella@gmail.com

**Keywords:** severity, score, coronavirus, SARS-COV-2, COVID-19, WHO, classification, prediction

## Abstract

**Objectives:** Assess the predictive accuracy of the WHO COVID-19 severity classification on COVID-19 hospitalized patients. The secondary aim was to compare its predictive power with a new prediction model, named COVID-19 EPI-SCORE, based on a Bayesian network analysis. **Methods:** We retrospectively analyzed a population of 295 COVID-19 RT-PCR positive patients hospitalized at Epicura Hospital Center, Belgium, admitted between March 1st and April 30th, 2020. **Results:** Our cohort’s median age was 73 (62–83) years, and the female proportion was 43%. All patients were classified following WHO severity classification at admission. In total, 125 (42.4%) were classified as *Moderate*, 69 (23.4%) as *Severe*, and 101 (34.2%) as *Critical*. Death proportions through these three classes were 11.2%, 33.3%, and 67.3%, respectively, and the proportions of critically ill patients (dead or needed Invasive Mechanical Ventilation) were 11.2%, 34.8%, and 83.2%, respectively. A Bayesian network analysis was used to create a model to analyze predictive accuracy of the WHO severity classification and to create the EPI-SCORE. The six variables that have been automatically selected by our machine learning algorithm were the WHO severity classification, acute kidney injury, age, Lactate Dehydrogenase Levels (LDH), lymphocytes and activated prothrombin time (aPTT). Receiver Operation Characteristic (ROC) curve indexes hereby obtained were 83.8% and 91% for the models based on WHO classification only and our EPI-SCORE, respectively. **Conclusions:** Our study shows that the WHO severity classification is reliable in predicting a severe outcome among COVID-19 patients. The addition to this classification of a few clinical and laboratory variables as per our COVID-19 EPI-SCORE has demonstrated to significantly increase its accuracy.

## 1. Introduction

Coronavirus Disease 2019 (COVID-19) [1,2] is putting governments and respective healthcare systems under extraordinary stress.

The extreme facility of transmission of the disease with the incredibly wide range of clinical scenarios (ranging from asymptomatic carriers to critically ill patients) warrants extraordinary research efforts directed mainly to the discovery of one or more reliable vaccines or treatment. In this context, it is of great importance to ensure the validation of severity scores which can help to direct the choice of the best therapeutic options and which can help us to predict the prognosis of each patient presenting with a clinical diagnosis of COVID-19.

To date, we have learnt that the clinical spectrum of COVID-19 infection ranges from asymptomatic to life-threatening conditions. Patients with mild disease can present with symptoms of fever, cough, and fatigue [1] and some patients deteriorate quickly after a short period of mild symptoms. Sepsis, respiratory failure, acute respiratory distress syndrome (ARDS), heart failure, and septic shock are commonly observed in critically ill patients [2,3].

The early detection of patients at risk to develop critical illness may aid in delivering proper care and reduce mortality. Current literature has already identified several risk factors [4,5].

Meanwhile, many groups searched means to classify patients according to severity through scores and classification systems to help clinician in care and triage. Lian et al. proposed the COVID-GRAM score designed to predict critical illness among patients (dead, admitted to Intensive Care Unit (ICU), or mechanically ventilated) [6]. Ten variables were used in this score, giving an Area Under the Curve (AUC) of their Receiver Operating Characteristics (ROC) analysis of 0.88. Ji et al. developed the CALL (C = comorbidity, A = age, L = lymphocyte count, L = lactate dehydrogenase) score on data of 208 Chinese patients to predict disease progression. Their model used four variables and returned an AUC of 0.91 on their development cohort [7]. An American group proposed another score using 641 patients to predict either intensive care admission or mortality. Their score yielded an AUC of 0.74 for predicting ICU admission, and 0.82 for predicting mortality [8]. The well-known CURB-65 (C = Confusion, U = blood Urea nitrogen, R = Respiratory rate, 65 = age 65 or older) score and the Pneumonia Severity Index (PSI) have also been used on 681 laboratory-confirmed Turkish patients to predict mortality of COVID-19 related pneumonia with an AUC of 0.88 and 0.91, respectively [9]. All these scores were designed using multivariate regression models. 

The World Healthcare Organization (WHO) also has proposed a classification which separates patients affected by COVID 19 according to the gravity of their clinical scenario [10]. Given the scarcity of data on its predictive power in real life, we sought to assess it on a group of patients admitted with a diagnosis of COVID-19 to Epicura Centre Hospitalier, a healthcare facility located in the south of Belgium. We also sought to compare its predictive power with a new prediction model based on a Bayesian network analysis obtained on the same population (COVID EPI-SCORE).

Bayesian networks are powerful models that compactly represent the joint probability distribution defined by the set of variables under study. They are composed of “nodes” representing variables of interest, such as age or death, directed links representing direct probabilistic dependencies between the variables, and marginal and conditional probability distributions quantifying the probabilistic relationships between variables. Once fully specified, the Bayesian network is a powerful probabilistic expert system that can compute a score defined as the posterior probability of the target variable given evidence on a subset of variables [11].

## 2. Results

### 2.1. Characteristics of Patients Following WHO Severity Classification

Subsequent to our inclusion and exclusion criteria specified in the method section, our cohort consisted of 295 patients. During the hereabove mentioned time course, all admitted patients were RT-PCR COVID19 confirmed cases and obtained definite outcomes—i.e., dead or discharged alive. Our cohort’s median age was 73 (62–83) years, and the female proportion was 43%. All patients were classified following WHO severity classification at admission (Appendix A and Appendix B); 125 (42.4%) were *Moderate*, 69 (23.4%) were *Severe*, and 101 (34.2%) were *Critical*. Mild patients were not hospitalized; thus, this class was not represented in our study. Death proportions through these three WHO severity classes were 11.2%, 33.3%, and 67.3% (Appendix B1), respectively. The proportions of critically ill patients, defined by either dead or underwent invasive mechanical ventilation (IMV), were 11.2%, 34.8%, and 83.2%, respectively (Appendix B2). Death or Critical Illness (death or IMV) were the only variables with statistically significant differences between the three WHO severity groups after Bonferroni’s correction. Other variables showed overall group differences, but inter-group analysis demonstrated only partial differences. Hence, dyspnea and cough were more prevalent in *Severe* or *Critical* patients than *Moderate* patients (Appendix B3,4). *Severe* or *Critical* patients also presented lower pulsed oxygen measures compared to *Moderate* patients (Appendix B9). C-reactive protein (CRP) was also higher in *Severe* and *Critical* patients than in *Moderate* patients (Appendix B11). *Critical* patients did have higher body temperature, lower partial oxygen pressure and lower lymphocyte count (Appendix B7, B10, B12). They experienced more AKI, had a higher Lactate Dehydrogenase level (LDH), Creatinine Phosphokinase level (CPK), and Aspartate Aminotransferase (AST) enzyme titer and lower albumin (Appendix B6, B13, B13–16). They were more frequently taking Angiotensine Converting Enzyme (ACE) inhibitors than *Moderate* patients (Appendix B5).

Neither chronic conditions nor age or previous institutionalization in a nursing facility were significantly different between severity groups.

### 2.2. Bayesian Network Analysis and Score Modeling

**Figure 1:**
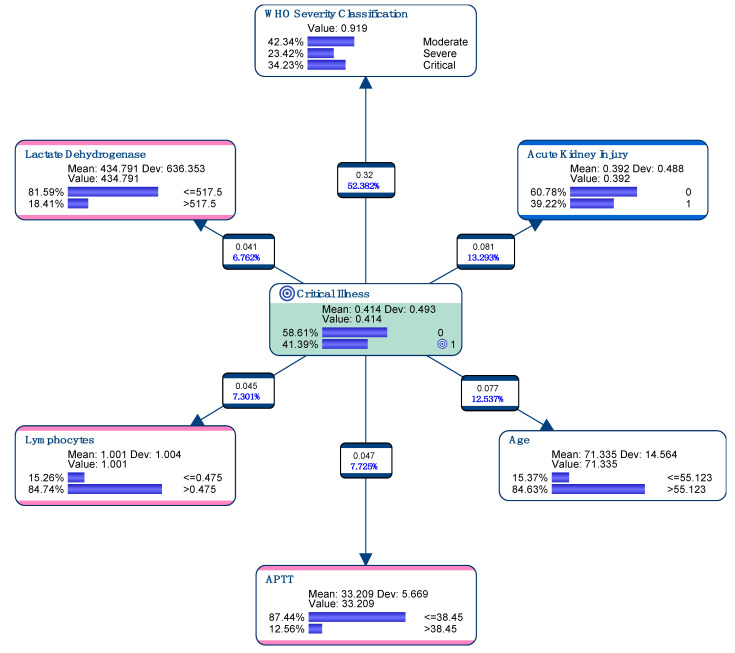
Graphical presentation of variables that have been automatically selected by our machine learning algorithm following the Target Node Critical Illness, giving the EPI-SCORE. Dev.: Standard Deviation.

All 127 variables were analyzed to find the most compact model to predict Critical Illness (Target Node)—i.e., death or need for IMV (Figure 1). In total, 122 (41.4%) of the patients were classified as suffering from Critical Illness following criteria developed in Method section. The six variables have been automatically selected by the machine learning algorithm to define our model: the WHO severity classification, acute renal failure, older age (>55 years), LDH Levels (>517Ui/L, low lymphocyte count (<0.475 × 1000/mm^3^) and prolonged aPTT (>38.45 s) at admission (Figure 1). This model was used to create the EPI-SCORE.

The 10-Fold Cross-Validation procedure allowed us to estimate that we can expect our Machine-learning workflow to generate a model with a minimum ROC index of 86.3% when evaluated on unseen patients (Table 1).

**Figure 2:**
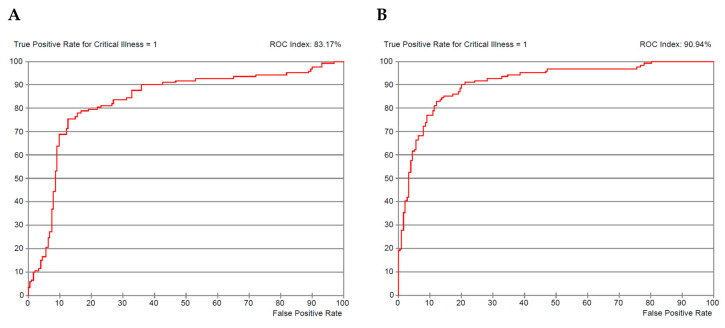
ROC Curves of Bayesian Network Model. ROC curve of WHO severity classification (**A**) and COVID-19 score (EPI-SCORE) (**B**).

ROC curve index obtained by this model learned and evaluated through the entire set of patients was 83.8% and 91% for the one based on WHO classification only and our EPI-SCORE (all six selected variables), respectively (Figure 2A,B).

## 3. Discussion

The present study offers a thorough analysis of the COVID-19 severity classification proposed by the WHO. We used data describing laboratory-confirmed patients at hospital admission to help clinicians in early recognition of pejorative evolution of COVID-19-related pneumonia. The WHO classification did show good prognostic power for severity prediction throughout our cohort, confirmed by both conventional statistics and Bayesian network modeling.

Moreover, this study proposes a new and original severity predictive analysis through Bayesian network modeling. With the herein designed model, WHO severity classification could be strengthened by adding five other variables to help predict Critical Illness in SARS-COV-2 induced pneumonia patients to propose our COVID-19 EPI-SCORE. As far as we know, this is the first risk score developed using Bayesian Network Analysis based on a European cohort. This method proposes a powerful associative model that yielded a simple score based on the well-known WHO classification and was internally validated by 10-fold randomization. Besides being useful in clinical practice, this score could be valuable in the classification of severity of patients in clinical trials.

The use of a nonparametric Bayesian network has several advantages over classical regression: variables can be categorical or numerical, relationships can be linear or non-linear, missing values are automatically processed when learning the network. Moreover, as it is a Bayesian model, we can use the score without having all the information on the model’s variables; the Bayes theorem allows us to update the output incrementally upon reception of pieces of evidence describing the patient.

We chose to adopt the WHO severity classification to conduct our analysis as it is a worldwide referenced institution. This classification was designed on clinical, radiological, and biological parameters with clinical management propositions for each of the four levels of severity. Mild patients were not hospitalized and thus not represented in our study. As far as we know, our model is the first to predict pejorative clinical evolution through this classification and could, therefore, participate in making it more relevant in clinical practice.

Indeed, our analysis demonstrated substantial differences in Critical Illness (*p* < 0.001) (dead or need of IMV) when using WHO’s proposed severity groups (10-folded ROC index of 83.8%). The Bayesian network could increase sensitivity and specificity to predict Critical Illness by adding age, AKI, LDH levels, lymphocyte count, and prolonged aPTT (10-folded ROC index of 86.3%). The EPI-SCORE thereby created is publicly available at the following address: https://simulator.bayesialab.com/#!simulator/108795509568.

The model outputs the posterior probability of suffering from Critical Illness (ranging from 0 to 1). The initial value of this probability (the prior) is the prevalence observed in our cohort (0.41). It is updated after each piece of evidence on the patient. The slider “Your Prior” eventually allows to modify estimated prevalence of Critical Illness.

Age, LDH levels and lymphocyte count are variables known to be predictive of poor outcome and already proposed in other scores, such as the COVID-GRAM score [6], the CALL score [7] or Zhao et al. risk score [8] and other studies predicting disease severity [12].

Interestingly, AKI appeared to be relevant in our analysis, while other scores do not include altered kidney function. However, several studies pointed out the association of kidney injury at admission with pejorative outcomes [2,13,14].

Moreover, prolonged aPTT can be an indicator of the well-described COVID19 coagulopathy triggered by the acute phase cytokine storm [15]. Likewise, in a retrospective study by Tang et al., encompassing data from 183 consecutive patients with COVID-19, non-survivors had significantly prolonged PT and aPTT compared with survivors at initial evaluation [16].

Given that WHO classification includes parameters of respiratory failure, such as respiratory rate, oxygen pulsed saturation, the need for oxygen, the presence of ARDS, and septic shock, our new score can be considered as very complete in regard to the spectrum of known predictive factors of pejorative outcomes.

Meanwhile, other variables were also associated with severity through our Bayesian analysis, but variables retained by our model are of greater interest as they are independently associated with our outcome. For example, the Bayesian association model with Critical Illness as Target Node showed a very strong association between age and comorbidities; thus, these two variables could not be both represented in the final score. Age and comorbidity burden have indeed already been demonstrated to be predictive of severity and mortality [17] and used through different score propositions like the ones mentioned above.

Cardinal symptoms were previously described as fever, cough, and dyspnea [1]. Patients presenting with cough and dyspnea in this study were indeed more severely ill.

Anosmia and dysgeusia were noted in only 6.4% of all patients included in our study. This represents a marked reduction compared to the data published already in the literature [18,19]. The low frequency of anosmia and dysgeusia is probably due to the fact that these symptoms are very difficult to objectify, in particular in patients with fever, cough and dyspnea, which were the most prevalent symptoms at admission. It is therefore possible that their rate is under-estimated.

C-reactive protein, significantly associated with more severe illness in our analysis, was also described as predictive of poor outcome [20].

Lastly, we pointed out that patients taking ACE inhibitors were also more prone to be severely ill. Several explanations have surfaced through literature, including the role of ACE as severe acute respiratory distress syndrome (SARS) virus receptor. However, no objective study has shown until today, the definite role of ACE inhibitors intake as a formal predictor of Critical Illness [12,21]. However, no objective study was shown until today, through the definite role of ACE inhibitor intake as a formal predictor of critical illness [12,21].

Our study has some limitations. The most significant one is its retrospective nature, which makes the final assumptions weaker than if the study were run in a prospective fashion. A general limitation common to most scores developed for COVID-19 infection is that they require a blood sample and imaging and therefore could be used only in patients already admitted in the hospital. The model has been designed to create a score in hospitalized patients. Obviously, having been structured and built upon a population of patients with a median age of 73-years-old, it applies to a population with these characteristics. To understand if it is applicable to a different subset of patients (i.e., younger patients), it should be used prospectively in a different trial and this is our goal for future research efforts.

EPI-SCORE has been shown to be more reliable than the WHO Severity Score in predicting severe outcomes in patients hospitalized for a COVID-19 infection. The fact that it includes the WHO classifiaction could be interpreted at first sight as a limitation. In reality, the EPI-SCORE is not intended to substitute the WHO Score but to increase its robustness and reliability in clinical practice. Therefore, our hope is that the two scores could be used in conjunction, with the aim to help clinicians to assign to each patient a prognosis and a line of treatment adequate to it.

Another limitation of our study was that unfortunately data could be missing in anamnestic information, especially in secondary symptoms, such as anosmia, that could be less invalidating in comparison to cardinal symptoms, such as fever and dyspnoea, in patients arriving in settings of respiratory distress at the emergency department. Moreover, respiratory rate was lacking in 172 patients, thus moderate and critical classes could be underestimated as these patients were assimilated as having a respiratory rate of less than 30. As such, their classification was made upon the other criteria.

## 4. Materials and Methods

### 4.1. Study Population

We retrospectively analyzed a population of 355 patients hospitalized at Epicura Hospital Center (Province of Hainaut, Belgium), between March 1st and April 30th, 2020, who tested positive for Severe Acute Respiratory Syndrome related to coronavirus-2 (SARS-COV-2) using real-time reverse transcriptase-polymerase chain reaction (RT-PCR) on a nasopharyngeal swab.

**Figure 3:**
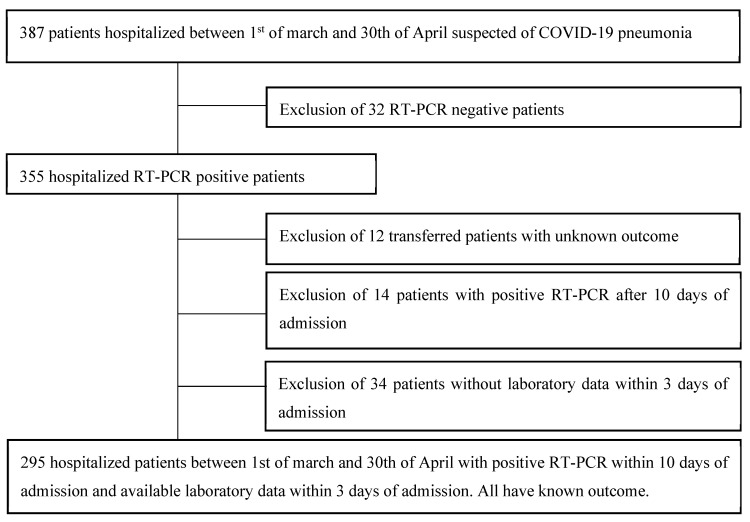
Flowchart of patient selection.

In an effort to consider only patients with community acquired COVID-19 disease, we only included patients who tested positive before the 10th day of admission in our hospital (14 patients with positive RT-PCR after 10 days of admission were thus excluded). Those whose laboratory tests were not available within the first 3 days of admission in our COVID19 ward were excluded (34 patients). Secondary transferred patients with unknown outcome were also excluded (12 patients). A total of 295 patients was considered therefore eligible for our study (Figure 3).

The local Ethics Committee approved the study protocol (EpiCURA-OM034, P2020/016) and waived the need for informed consent. We performed the study following the ethical standards of the 1964 Declaration of Helsinki and its later amendments. The date of the last follow-up was July 10th, 2020, date on which all patients’ primary outcomes—i.e., death or discharged alive—were obtained.

### 4.2. Patients’ Management and Severity Classification

Patients’ criteria of hospitalization were not standardized; patients with a suspicion of COVID-19 were admitted according to the emergency department physician’s clinical judgment along with an Infectious Disease Specialist agreement. Mostly, patients were admitted to stay at the hospital when they showed any signs of respiratory failure (need for oxygen, high respiratory rate, low pulse oximetry, altered consciousness) or showed any complication needing close monitoring (i.e., acute kidney injury (AKI), dehydration, fall, confusion, anemia). Medical treatment was decided according to the treating physician and the Infectious Diseases specialist and included oral hydroxychloroquine (400 mg twice a day orally on the first day followed by 200 mg twice a day orally until day 5) alongside conventional symptomatic treatment.

COVID-19 infection severity was determined and defined as *Moderate*, *Severe*, or *Critical* according to the WHO severity classification, last updated at the end of May 2020 [10]. *Critical* COVID-19 patients corresponded to patients with bilateral, pathognomonic radiographic findings of COVID-19 pneumonia in association with either septic shock or ARDS according to Berlin’s criteria [22], defined in the first 72 h of hospital admission. *Severe* COVID-19 patients corresponded to patients with pathognomonic radiographic findings of COVID-19 pneumonia with the need for oxygen at admission, saturation <90% on room air or respiratory rate above 30/min: Lastly, *Moderate* COVID-19 patients corresponded to patients presenting with radiographic findings of pneumonia without the hereabove mentioned clinical findings. AKI was defined following Acute Kidney Injury Network criteria [23] and was used as follows: absolute increase in serum creatinine above 0.3 mg/dL or increase in serum creatinine above 1.5 times the baseline in the first 72 h of hospital admission.

To analyze the predictive power of the WHO classification, we considered as an outcome of reference “Criticall Ilness” as the need for mechanical ventilation during the hospital stay or death. Furthermore, we decided to design a new score based on this outcome and we compared its predictive accuracy with the WHO severity classification.

### 4.3. Data Collection

Data were retrospectively extracted from the Epicura electronic medical records by the investigators and anonymized. Information about demographics, chronic medical conditions, and medications was gathered alongside the Charlson Comorbidity Index [10] to take the patient’s comorbidity into account and its effect on short-term mortality.

Presenting symptoms, vital parameters, clinical signs, laboratory findings, and arterial blood gas, and lung imaging abnormalities were also collected at admission.

### 4.4. Conventional Statistical Analysis

Descriptive statistics were computed for all study variables according to WHO classification. Categorical variables were expressed as count (percentage) and continuous variables as median (25–75th percentiles), as appropriate. Differences between severity groups—i.e., *Moderate, Severe,* and *Critical*—were assessed using a Chi-square test with post hoc Bonferroni correction for categorical variables and Kruskal–Wallis test with post hoc pairwise comparison and Bonferroni correction for continuous variables. In determining the predictive power of WHO severity score and of our new survival score, a non-parametric Receiver operating curve (ROC) model was used.

A threshold of *p*-value of 0.05, after correction, was used to define statistically significant tests. Statistical analyses were performed using the SPSS 26.0 (SPSS, Inc., Chicago, IL, USA).

### 4.5. Bayesian Network Analysis and Score Modeling

Bayesian networks are models that include a qualitative part, Directed Acyclic Graphs (DAG), to indicate the dependencies, and a quantitative part, local probability distributions, to specify the probabilistic relationships. In a DAG, the nodes are the variables of interest, such as age or death, whereas the directed links represent statistical dependencies between the variables.

The local probability distributions are either marginal, for nodes without incoming links, or conditional, for nodes with incoming links. In this case, the dependencies are quantified by conditional probability tables (CPT) for each node given the nodes associated with the incoming links in the graph. Once fully specified, a Bayesian network compactly represents the joint probability distribution (JPD) defined by the set of all variables (here 127 variables). Thus, we can use it to compute the posterior probabilities of any variables given evidence about any other subset.

The Bayesian network presented in this study has been machine-learned with the Augmented Markov Blanket algorithm implemented in BayesiaLab (Bayesia S.A.S., Changé, France). This algorithm uses the Minimum Description Length (MDL) score to find the set of variables that allows for making the Target Node (Critical Illness) conditionally independent of all the other variables. The MDL score manages the trade-off between complexity and information, allowing arcs only when the reduction in uncertainty offsets the additional cost in model representation.

As per the continuous variables, our supervised learning algorithm chose the number of thresholds and their values to maximize the Mutual Information with our Target node.

We used a 10-Fold Cross-Validation approach to assess the quality of the learned network (Table 1). In this method, we randomly split the dataset into ten equal-sized subsets of patients. We then conducted learning by using nine subgroups of patients and evaluate the performance of the obtained model on the remaining subset. This workflow is repeated ten times to use all the data for learning and testing, but never simultaneously.

## 5. Conclusions

This study was able to evaluate the predictive accuracy of the COVID-19 WHO severity classification. We demonstrated that this classification is a reliable and accurate clinical tool able to consistently predict a critical outcome among COVID-19 patients. The addition of a few simple clinical and laboratory variables to this classification as per our COVID-19 EPI-SCORE has shown to significantly increase its accuracy. Multicenter studies are warranted to confirm our preliminary data. The EPI-SCORE is intended to implement the current WHO Severity SCORE in the acute setting. Thanks to its high predictive accuracy, we believe it can be used first of all by those clinicians who stand in the front line, helping them to make appropriate decisions during admissions of patients to COVID-19 dedicated wards. Assigning each patient to a very specific category of risk as easily by adopting the EPI-SCORE can also help physicians in hospital wards to guide the therapies (if available) and to tailor the healthcare allied personnel commitment, hopefully reducing the continuous and harmful waste of resources. Finally, we believe that the EPI-SCORE could also be used to select the patients for future randomized trials in a more objective fashion, according to their prognosis.

## Figures and Tables

**Table 1:** Model performance 10-Fold.

K-Fold (10), Structural Priors, Target: Critical Illness		
**Value**	0	1
**Gini Index**	29.66%	42.53%
**Relative Gini Index**	72.30%	72.14%
**Lift Index**	1.41	1.65
**Relative Lift Index**	92.45%	88.18%
**ROC Index**	86.39%	86.31%
**Calibration Index**	57.16%	58.90%
**Binary Log-Loss**	0.46	0.46

## Appendix A. Patients’ Characteristics According to Severity Classification following WHO-Guidelines

		**Moderate (n = 125)**	**Severe (n = 69)**	**Critical (n = 101)**	**Total (n = 295)**	***p*-Value**
Demographic data						
	age	72 (60; 83)	75 (62; 86)	72 (66; 82)	73 (62; 83)	0.377
	female	56a, b (44.8%)	37b (53.6%)	35a (34.7%)	128 (43.4%)	0.045
	dead	14a (11.2%)	23b (33.3%)	68c (67.3%)	105 (35.6%)	0.000
	Critical Illness (dead or IMV)	14a (11.2%)	24b (34.8%)	84c (83.2%)	122 (41.4%)	0.000
	Institutionalized	29 (23.8%)	20 (29.4%)	27 (26.7%)	76 (26.1%)	0.687
Symptoms						
	Fever	76 (61.3%)	48 (73.8%)	72 (72.0%)	196 (67.8%)	0.116
	Cough	51a (41.5%)	47b (72.3%)	59b (59.0%)	157 (54.5%)	0.000
	Dyspnea	49a (40.2%)	45b (71.4%)	70b (70.7%)	164 (57.7%)	0.000
	Thoracic pain	12 (9.8%)	2 (3.2%)	4 (4.1%)	18 (6.4%)	0.114
	Dysgeusia or Anosmia	6 (5.0%)	5 (7.9%)	7 (7.2%)	18 (6.4%)	0.679
	Myalgia	17 (14.0%)	13 (20.6%)	22 (22.7%)	52 (18.5%)	0.234
	Headache	6 (5.0%)	9 (14.3%)	10 (10.3%)	25 (8.9%)	0.090
	Nausea	14 (11.5%)	8 (12.9%)	8 (8.2%)	30 (10.7%)	0.605
	Vomiting	15a (12.2%)	5a, b (7.8%)	2b (2.0%)	22 (7.7%)	0.017
	Abdominal pain	15 (12.3%)	3 (4.7%)	5 (5.1%)	23 (8.1%)	0.077
	Confusion	19 (15.4%)	9 (13.8%)	18 (18.0%)	46 (16.0%)	0.759
	Fall	22 (17.9%)	5 (7.6%)	12 (12.1%)	39 (13.5%)	0.125
	Diarrhoea	15 (12.3%)	5 (7.8%)	13 (13.0%)	33 (11.5%)	0.563
Chonic conditions						
	Mininum of one comorbidity	111 (88.8%)	60 (87.0%)	95 (94.1%)	266 (90.2%)	0.248
	History of heart infarction	24 (19.4%)	8 (11.8%)	19 (18.8%)	51 (17.4%)	0.373
	History of heart failure	18 (14.4%)	11 (15.9%)	21 (20.8%)	50 (16.9%)	0.430
	History of peripheric arterial disease	12 (9.6%)	6 (8.7%)	11 (10.9%)	29 (9.8%)	0.889
	History of stroke or transient ischemic attack	10 (8.0%)	10 (14.5%)	18 (17.8%)	38 (12.9%)	0.082
	History of cerebro-vascular disease	43 (34.4%)	25 (36.2%)	43 (42.6%)	111 (37.6%)	0.435
	Dementia	18 (14.4%)	13 (19.1%)	21 (20.8%)	52 (17.7%)	0.429
	COPD	13 (10.4%)	14 (20.9%)	13 (12.9%)	40 (13.7%)	0.125
	Asthma	12 (9.6%)	8 (11.9%)	7 (6.9%)	27 (9.2%)	0.536
	Chronic liver disease	4a (3.2%)	9b (13.4%)	4a, b (4.0%)	17 (5.8%)	0.009
	Diabetes (type I or II)	41 (32.8%)	19 (27.5%)	33 (32.7%)	93 (31.5%)	0.717
	Dyslipidemia	42 (33.6%)	29 (42.6%)	47 (46.5%)	118 (40.1%)	0.127
	Hypertension	75 (60.0%)	48 (70.6%)	68 (67.3%)	191 (65.0%)	0.280
	Past or active smoker	25 (22.1%)	11 (17.7%)	26 (27.7%)	62 (23.0%)	0.339
	Chronic kidney failure	31 (24.8%)	15 (21.7%)	24 (23.8%)	70 (23.7%)	0.891
	CHARLSON	4 (2; 6)	5 (3; 6)	5 (3; 6)	5.0 (2.0; 6.0)	0.259
Chronic medications						
	Immunosuppresive treatment	6 (4.9%)	6 (8.7%)	9 (8.9%)	21 (7.2%)	0.433
	Oral antidiabetics	26 (20.8%)	9 (13.0%)	23 (22.8%)	58 (19.7%)	0.268
	Insuline	17 (13.6%)	6 (8.7%)	15 (14.9%)	38 (12.9%)	0.476
	Antiplatelet therapy	39 (31.2%)	19 (27.5%)	43 (42.6%)	101 (34.2%)	0.082
	Anticoagulation therapy	26 (20.8%)	17 (24.6%)	20 (19.8%)	63 (21.4%)	0.737
	ACE inhibitor	16a (12.8%)	15a, b (21.7%)	28b (27.7%)	59 (20.0%)	0.019
	Sartan	17 (13.6%)	6 (9.0%)	12 (11.9%)	35 (11.9%)	0.639
Admission vitals						
	Temperature (℃)	37.2a (36.4; 38)	37.5a, b (36.7; 38.2)	37.8 b (36.8; 38.3)	37.5 (36.6; 38.2)	0.044
	Heart rate (/min)	90 (78; 102)	87.5 (75; 102)	91 (80; 100)	90 (78; 101)	0.751
	Systolic blood pressure (mmHg)	136.5 (120; 150)	135.5 (117; 154)	130.5 (118; 152)	133 (120; 150)	0.740
	Diastolic blood pressure (mmHg)	76 (70; 87)	75 (66; 86)	73 (65; 85)	75 (67; 86)	0.263
	Need of oxygen at admission	0a (0%)	31b (44.9%)	31b (30.7%)	62 (21%)	0.000
	Pulse oxymetry	95a (93; 97)	89b (88; 94)	91b (85.5; 94)	93 (90; 96)	0.000
Arterial blood gas analysis						
	pH	7.48 (7.44; 7.50)	7.47 (7.41; 7.53)	7.47 (7.43; 7.50)	7.47 (7.43; 7.50)	0.844
	paCO2 (mmHg)	33 (30; 37)	35 (31; 43)	34 (29; 37)	34 (30; 38)	0.120
	paO2 (mmHg)	67 a (60; 74)	64 a, b (54; 72)	61b (54; 70)	64 (56; 73)	0.030
	Bicarbonate (mmol/ L)	25.5a, b (22.8; 27.2)	26.3a (23.5; 30.1)	24.0b (21.3; 27.1)	25.3 (22.5; 27.2)	0.014
	Lactates (mmol/L)	1.2 (0.8; 1.6)	1.4 (0.9; 2.2)	1.4 (1.0; 2.0)	1.3 (0.9; 1.9)	0.136
Laboratory work-up	C-reactive protein mg/L	61.7a (23.8; 105.8)	90.7b (45.5; 142.3)	92.8b (53.4; 153.1)	81.3 (39.4; 132.8)	0.001
	Hemoglobin g/dL	13.5 (11.9; 14.9)	13.6 (12.7; 14.7)	13.5 (12,0; 15,0)	13,6 (12,2; 14,8)	0,843
	Platelets × 1000/mm^3^	222 (160; 290)	203 (147; 263)	191 (154; 251)	204 (154; 268)	0.110
	White blood cell count × 1000/mm^3^	7.2 (4.7; 10.5)	7.0 (5.8; 9.9)	6.8 (5.4; 8.3)	7.0 (5.2; 9.4)	0.281
	Neutrophiles × 1000/ mm^3^	5.7 (3.4; 8.1)	5.2 (4.3; 8.4)	5.5 (3.9; 6.9)	5.5 (3.7; 7.5)	0.554
	Lymphocytes × 1000/mm^3^	0.9a (0.7; 1.2)	0.8a, b (0.5; 1.2)	0.7b (0.5; 1.1)	0.8 (0.6; 1.2)	0.033
	Neutrophiles/lymphocytes	6.3 (3.2; 10.3)	7.2 (3.7; 14.3)	6.6 (3.8; 11.1)	6.8 (3.6; 11.7)	0.275
	Urea mg/dL	40 (27; 58)	41 (26; 78)	47 (34; 75)	43 (29; 67)	0.110
	Creatinine mg/dL	1.0 (0.8; 1.4)	1.0 (0.8; 1.6)	1.1 (0.9; 1.6)	1.1 (0.9; 1.5)	0.259
	Urea/creat	38 (29; 53)	37 (29; 52)	42 (32; 52)	39 (30; 52)	0.159
	Acute kidney failure in 72 h of admission	34a (27.2%)	25a, b (36.8%)	56b (55.4%)	115 (39.1%)	0.000
	Sodium mmol/L	137 (135; 140)	138 (134; 140)	138 (134; 140)	138 (134; 140)	0.591
	Potassium mmol/L	4.1 (3.7; 4.4)	4.0 (3.6; 4.4)	4.0 (3.7; 4.5)	4.0 (3.7; 4.4)	0.721
	Chloride mmol/L	97a,b (94; 100)	96a (93; 99)	98b (94; 101)	97 (94; 100)	0.034
	Bicarbonate mmol/L	25 (22; 27)	25 (23; 27)	24 (22; 27)	25 (22; 27)	0.206
	Lactate dehydrogenases UI/L	317a (255; 395)	341a,b (284; 410)	421b (287; 548)	342 (270; 447)	0.000
	Creatinine phosphokinases UI/L	102a (53; 242)	111a,b (50; 330)	187b (90; 490)	142 (62; 344)	0.003
	Total bilirubin mg/dL	0.5 (0.4; 0.9)	0.5 (0.4; 0.7)	0.5 (0.4; 0.7)	0.5 (0.4; 0.8)	0.443
	Direct bilirubin mg/dL	0.3 (0.2; 0.4)	0.2 (0.2; 0.4)	0.3 (0.2; 0.4)	0.3 (0.2; 0.4)	0.587
	Aspartate aminotransferase (AST) UI/L	37a (26; 52)	37a,b (25; 63)	46b (31; 72)	40 (27; 62)	0.009
	Alanine aminotransferases (ALT) UI/L	27 (19; 40)	26 (16; 43)	26 (18; 45)	26 (18; 42)	0.873
	Gamma-glutamyl-transferases (gGT) UI/L	50 (32; 102)	59 (26; 99)	43 (29; 74)	48 (30; 92)	0.364
	Alkaline phosphatases UI/L	82a (58; 108)	79a (61; 105)	69b (52; 84)	77 (58; 97)	0.003
	INR	1.2 (1.1; 1.3)	1.1 (1.1; 1.2)	1.2 (1.1; 1.3)	1.2 (1.1; 1.3)	0.314
	Prothrombin time (seconds)	79 (68; 86)	81 (72; 91)	77 (68; 88)	79 (68; 88)	0.324
	Partial thromboplastin time (seconds)	32 (30; 35)	32 (30; 36)	33 (30; 36)	33 (30; 35)	0.314
	Albumin (mg/dL)	35.1a (32.0; 38.4)	33.4a,b (30.8; 37.3)	33.4b (30.4; 36.2)	34.3 (31.4; 37.6)	0.029
a, b, c: each letter denotes a subset of WHO Severity Classification categories whose column proportions do not differ significantly from each other at the 0.05 level after Bonferroni’s correction.						
